# Anomalous Right Coronary Artery in the Setting of Active Tuberculosis: A Multidisciplinary Management Challenge

**DOI:** 10.3390/life15050736

**Published:** 2025-05-01

**Authors:** Ana Peruničić, Matija Furtula, Stefan Veljković, Jovana Lakčević, Armin Šljivo, Valentina Balint, Slobodan Tomić, Sanja Vučinić, Milovan Bojić, Aleksandra Nikolić

**Affiliations:** 1 Cardiovascular Institute “Dedinje’’, 11040 Belgrade, Serbiafurtulam1205@gmail.com (M.F.); jovana.lakcevic@gmail.com (J.L.); valentinabalintjovanovic@gmail.com (V.B.); bobantomic99@gmail.com (S.T.); sanjakosticvucinic@gmail.com (S.V.); dedinje@ikvbd.com (M.B.);; 2 Department for Cardiosurgery, Clinical Center of University of Sarajevo, 71000 Sarajevo, Bosnia and Herzegovina; 3 Faculty of Medicine, University of Banja Luka, 78000 Banja Luka, Bosnia and Herzegovina; 4 Faculty of Medicine, University of Belgrade, 11000 Belgrade, Serbia

**Keywords:** ARCAPA, anomalous coronary artery, tuberculosis, coronary angiography, stress MRI, conservative treatment, case report

## Abstract

Anomalous origin of the right coronary artery from the pulmonary artery (ARCAPA) is a rare congenital coronary anomaly, with an uncertain prevalence and often diagnosed incidentally. This case report presents a 62-year-old male with ARCAPA diagnosed during an evaluation for chest surgery. The patient had a history of colon cancer and active tuberculosis, complicating the clinical management. He reported chest pain, shortness of breath, and palpitations, with atrial fibrillation observed on a 24 h Holter ECG. Coronary angiography revealed robust collateral circulation and a suspected anomalous origin of the right coronary artery, confirmed by CT imaging. The patient’s stress MRI showed mildly reduced left and right ventricular ejection fractions and perfusion deficits in the apical segments (2/17) of the septal and inferior walls. Given the patient’s comorbidities, including active tuberculosis, the Heart team decided on a non-operative management approach, focusing on careful monitoring and pharmacological management rather than immediate surgery. This case emphasizes the complexity of managing ARCAPA in the context of significant comorbidities, highlighting the importance of individualized, multidisciplinary treatment strategies. Early diagnosis using advanced imaging techniques is crucial, and a non-operative approach can be considered in patients with preserved left ventricular function and no significant ischemia, as demonstrated in this case.

## 1. Introduction

The anomalous origin of the right coronary artery from the pulmonary artery (ARCAPA) is a form of coronary artery malformation, occurring in only 0.002% of patients undergoing coronary angiography, representing a mere 0.12% of anomalies within this domain [1,2]. Left-to-right collaterals typically exist, facilitating oxygenated blood flow from the left coronary system into the territory of the right coronary artery (RCA). Regardless of sufficient collateralization, patients may experience symptoms attributed to “coronary-steal” as blood diverts into the lower-pressure pulmonary circulation [3], such as stable angina, dyspnea, and heart failure. Additionally, the condition carries a risk of sudden cardiac death, often attributed to fatal arrhythmias or ischemia-related complications [4]. Symptom onset typically occurs later in life, with most cases becoming clinically apparent between the ages of 40 and 60, although earlier detection may occur with advancements in imaging techniques and awareness of this rare anomaly [3].

The management of ARCAPA encompasses both operative and non-operative strategies, depending on the patient’s clinical presentation, age, symptoms, ventricular function, and evidence of myocardial ischemia. Operative strategies are considered the definitive treatment and are primarily aimed at revascularization of the RCA, either by direct reimplantation of the anomalous RCA into the aorta, thereby restoring a two-coronary artery system, or by performing a coronary artery bypass graft (CABG) if reimplantation is not feasible due to anatomical limitations [3]. Surgical correction eliminates the left-to-right shunt between the high-pressure left coronary system and the low-pressure pulmonary artery, reduces the risk of myocardial ischemia, and significantly decreases the risk of sudden cardiac death. Non-operative strategies are generally reserved for select asymptomatic patients, particularly those with normal biventricular function, no evidence of ischemia on stress testing, and a low risk profile. Management in such cases may involve medical therapy including anti-anginal medications or beta-blockers, as well as lifestyle modifications and close clinical follow-up with serial imaging and functional testing. In some cases, the coexistence of ARCAPA with comorbid conditions adds additional diagnostic and therapeutic challenges. For example, tuberculosis or metastatic disease—through its associated chronic inflammatory and granulomatous response—may exacerbate cardiovascular manifestations, including myocardial ischemia and arrhythmias [5,6].

Here, we present a case of ARCAPA successfully managed non-operatively in a 62-year-old male with extensive comorbidities. The aim of this report is to highlight the pivotal role of advanced diagnostic modalities, including coronary angiography, computed tomography, and stress imaging, in confirming the diagnosis of ARCAPA and delineating its clinical impact. Additionally, this report emphasizes the necessity of an individualized, multidisciplinary approach to treatment planning, particularly in patients with coexisting pathologies that significantly influence the risk–benefit analysis of surgical versus non-operative management strategies.

## 2. Case Presentation

A 62-year-old patient was referred to cardiology examination as part of the preparatory process for chest surgery. This decision was prompted by the identification of tumoral changes on a lung CT scan, raising concerns about a potential secondary deposit, especially in the context of the patient’s history of undergoing surgery for colon cancer two years prior. The patient reported chest pains and shortness of breath during physical exertion, along with palpitations. He was a former smoker (20 pack/year) and an occasional alcohol drinker. The patient’s previous ECG showed sinus rhythm with a heart rate of 57/bpm, normal axis, with one SVES and without necrosis, and changes in the T wave that led to the decision to send the patient for a 24 h Holter ECG, revealing paroxysms of atrial fibrillation with a response rate of 120/min, normal QRS complexes, and no definitive signs of ischemia, necrosis, or acute changes. The patient’s laboratory results revealed a hemoglobin level of 12.0 g/dL, a white blood cell count of 13.5 × 10^3^/μL, and a platelet count of 235 × 10^3^/μL. The neutrophil count was elevated at 82%, while lymphocytes were decreased to 10%, consistent with an acute infection. The basic metabolic panel showed sodium at 137 mEq/L, potassium at 3.9 mEq/L, chloride at 101 mEq/L, bicarbonate at 22 mEq/L, creatinine at 1.3 mg/dL, BUN at 20 mg/dL, and glucose at 97 mg/dL. Liver function tests showed an AST of 35 U/L, ALT of 40 U/L, alkaline phosphatase of 95 U/L, and a total bilirubin level of 0.9 mg/dL C-reactive protein (CRP) was elevated at 35 mg/L, indicating ongoing inflammation. Coagulation profile results included a prothrombin time of 14.1 s, an INR of 1.1, and an aPTT of 32.0 s. Cardiac biomarkers, including troponin I at 0.02 ng/mL (within normal limits, <0.03 ng/mL), CK, and CK-MB, were normal

**Figure 1:**
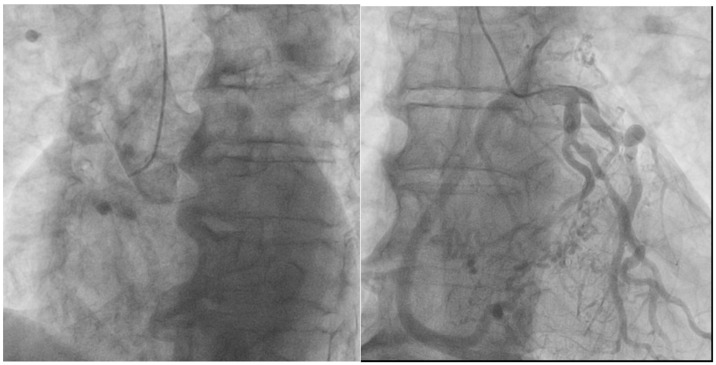
(**Left**): The right coronary artery is not visible from the right coronary sinus. (**Right**)*:* Robust collateral circulation observed between the left and right coronary arteries.

**Figure 2:**
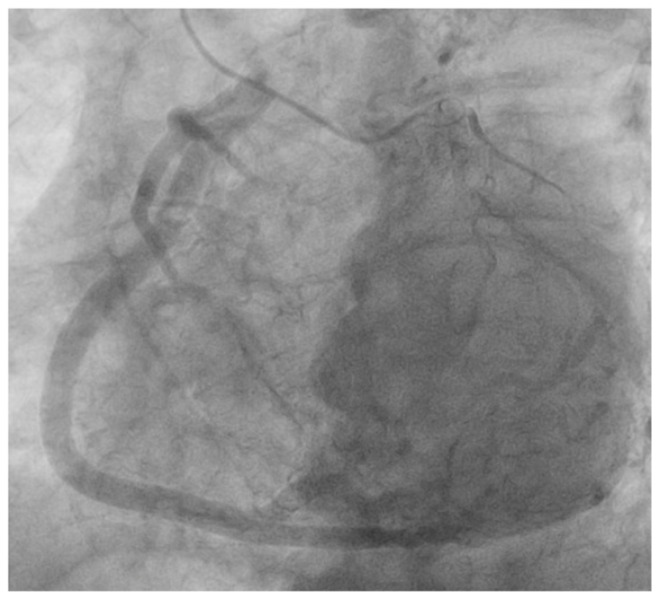
High likelihood that the right coronary artery has its origin in the pulmonary artery.

Due to confirmed atrial fibrillation with a rapid ventricular response on the ECG, a stress exercise test was not performed, leading to a referral for coronary angiography during which the ostium of the RCA was not located (Figure 1), but successful engagement of the left coronary artery (LCA) revealed a pronounced display of the RCA through robustly developed collaterals (Figure 1). This visual anomaly raised suspicion that RCA might have anomalous origin from the pulmonary artery (Figure 2).

**Figure 3:**
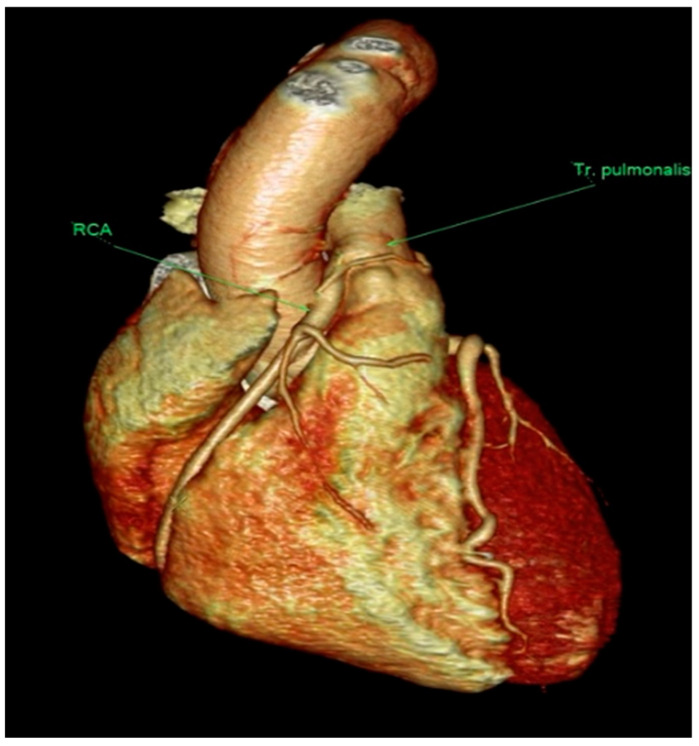
CT scan verification of the presence of ARCAPA syndrome.

Subsequent CT confirmation revealed the anomalous origin of RCA from the right anterolateral wall of the pulmonary artery (ARCAPA) (Figure 3).

Post-coronary angiography, additional investigations were carried out. Right heart catheterization indicated normal pressures in the pulmonary artery, a pulmonary capillary wedge pressure (PCWP) of 12 mmHg, a PA oxygen saturation of 75.8%, and a cardiac index (thermodilution method) of 3.38 L/min/m^2^.

**Figure 4:**
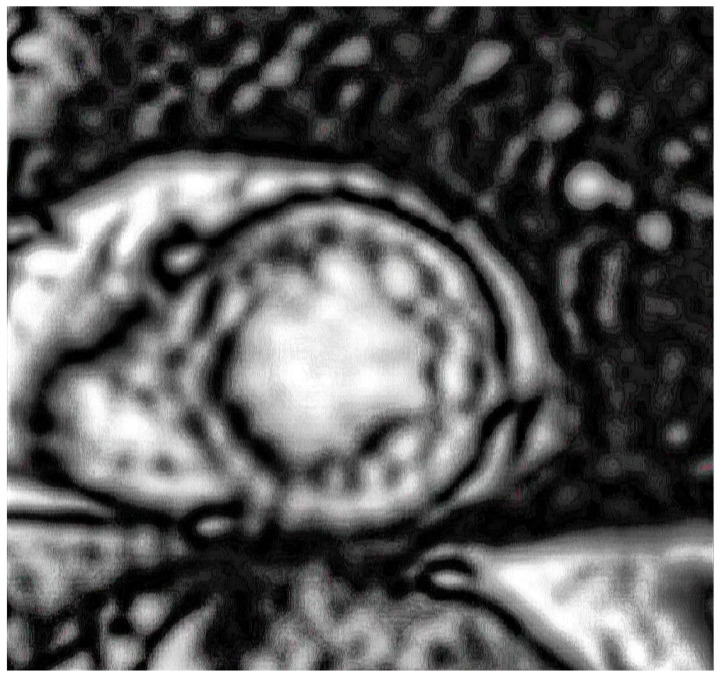
The stress MRI reveals a perfusion defect localized to the apical segments of both the septal and inferior walls.

The patient was scheduled for a planned surgical procedure, and it was advised that a stress MRI was conducted following the operation. The pathohistological examination of the tumor changes revealed the presence of a caseous granuloma, indicating an infection attributable to Mycobacterium tuberculosis. Stress MRI findings showed a normal-sized left (LV) and right (RV) ventricles with normal wall thickness and slightly reduced LV and RV ejection fractions (47% and 46%, respectively). Perfusion deficit, attributed to exertion, was noted in the apical segments of the septal and inferior wall (2/17 segments) (Figure 4). After presenting the patient to our Heart team, the decision was made to pursue non-operative treatment. Additionally, the patient was referred to the infectious disease specialist and oncologist. The initial approach focused on addressing the patient’s active tuberculosis, given its potential to complicate both pulmonary and cardiovascular conditions. The priority was to stabilize the pulmonary condition before making further decisions regarding ARCAPA management. The patient was informed of the surgical treatment options, but after discussing the risks and benefits with the Heart team, including consideration of his comorbidities, he actively participated in the decision to pursue non-operative treatment.

For non-operative management of ARCAPA, we started the patient on bisoprolol for rate control of atrial fibrillation, and SGLT-2 inhibitors to help improve heart failure symptoms and reduce the risk of further cardiac complications. Additionally, we added Rivaroxaban to reduce the risk of thromboembolic events. The patient was seen in clinic every 6 months for follow-up, with additional monitoring through echocardiograms to assess his cardiac function. Over the course of the follow-up period, the patient showed stable symptoms with no progression of ARCAPA-related ischemia, and his cardiac function remained stable. He did not experience worsening of symptoms related to tuberculosis, as his pulmonary condition improved with the completion of antibiotic/antituberculosis therapy. The patient’s outcome has been positive, with good management of both his cardiac and pulmonary conditions.

## 3. Discussion

This case report presents a rare anomaly of ARCAPA in a 62-year-old patient with significant comorbidities, including a history of colon cancer and active tuberculosis. Diagnostic evaluation confirmed ARCAPA through coronary angiography and CT imaging, with stress MRI revealing reduced biventricular ejection fractions and exertion-induced perfusion deficits. The presence of tuberculous granulomas further complicated the clinical scenario, necessitating a cautious approach. Ultimately, the Heart team opted for non-operative management, underscoring the need for individualized, multidisciplinary decision-making involving cardiology, invasive cardiology, and cardiac surgery in such complex presentations.

Several reports have documented successful non-operative management of ARCAPA in carefully selected patients with favorable clinical profiles. Due to the predominantly asymptomatic nature of ARCAPA, the true prevalence of this coronary anomaly remains uncertain, with only a limited number of documented cases presenting with significant clinical manifestations. The anomaly is often discovered incidentally during imaging or diagnostic procedures performed for unrelated conditions, and many patients remain asymptomatic or experience only mild symptoms. However, there have been rare instances in which ARCAPA presented alongside significant comorbidities such as atrial fibrillation, asthma, diabetes mellitus, and epilepsy, which can complicate the clinical picture and impact management decisions [7,8,9]. In one documented case, atrial fibrillation was the initial manifestation of ARCAPA, and although the patient declined surgery, short-term clinical stability was achieved with non-operative management at one-year follow-up [7]. Additionally, comorbidities such as asthma and epilepsy can complicate both the diagnostic process and treatment, given the potential overlap of symptoms like chest pain, shortness of breath, and altered mental status. Diabetes mellitus may further complicate the clinical scenario due to its association with accelerated atherosclerosis and potential for worsened cardiovascular outcomes [8,9]. These coexisting conditions necessitate a comprehensive, multidisciplinary approach to management and highlight the need for further research to better understand the clinical implications and true prevalence of ARCAPA in patients with such comorbidities.

Current guidelines for the management of patients with anomalous origin of coronary arteries from the pulmonary artery are primarily derived from expert consensus and are characterized by a low level of scientific evidence, necessitating further high-quality research to establish more definitive treatment protocols [10,11]. According to the latest European Society of Cardiology (ESC) Guidelines, surgery is recommended in patients with symptoms attributable to anomalous coronary artery and the surgery should be considered in asymptomatic patients with ventricular dysfunction, or myocardial ischemia attributable to coronary anomaly [12]. In patients with normal left ventricular ejection fraction and without ischemia, a non-operative strategy can be considered. Although our patient had symptoms and signs of myocardial ischemia, considering comorbidities such as prior colon cancer and active TBC infection, our Heart team made the decision of non-operative treatment with future follow-up.

The non-operative treatment strategy in this case was deemed appropriate due to the complex interplay of the patient’s comorbidities, particularly the history of colon cancer and active TBC infection, which both increase the risk associated with surgical intervention. A non-operative approach, involving careful monitoring, pharmacological management, and follow-up, ensures that the patient’s condition is assessed in a controlled manner, allowing for intervention only if necessary while avoiding the potential risks of surgery in a clinically vulnerable individual. This strategy also enables timely reassessment of the patient’s clinical status, particularly as the management of tuberculosis and any recurrence of colon cancer may alter the treatment trajectory.

## 4. Conclusions

ARCAPA is a rare anomaly with uncertain prevalence, often diagnosed incidentally. Management requires a personalized, multidisciplinary approach, particularly in patients with significant comorbidities like colon cancer and active tuberculosis. While surgery is recommended for symptomatic patients, a non-operative approach is appropriate for those with preserved left ventricular function, as shown in this case. Timely diagnosis and individualized treatment are crucial for optimizing outcomes in such complex cases.

## Data Availability

Data are available upon request.
